# Uncovering the culprit of recurrent hemoptysis: A case report of bronchial Dieulafoy disease

**DOI:** 10.1097/MD.0000000000041787

**Published:** 2025-03-14

**Authors:** Jiabin Qian, Yiheng Qian, Ruilin Chen, Xin Lv

**Affiliations:** a Department of Pulmonary Medicine, The First Affiliated Hospital of Zhejiang Chinese Medical University (Zhejiang Provincial Hospital of Chinese Medicine), Hangzhou, China.

**Keywords:** bronchial artery embolization (BAE), bronchial Dieulafoy disease (BDD), bronchoscopy, hemoptysis

## Abstract

**Rationale:**

Bronchial Dieulafoy disease (BDD) is caused by vascular malformations in the bronchial wall, which may rupture and bleed spontaneously or due to external factors. Bronchial artery embolization (BAE) is the treatment of choice.

**Patient’s concerns:**

The patient in this case had a 15-year history of recurrent hemoptysis, which persisted despite aggressive medical treatment. Due to a stent in the iliac artery, conventional transfemoral BAE was not feasible.

**Diagnoses:**

Fiberoptic bronchoscopy revealed exposed mucosal vessels with vascular malformations in the left upper lobe. Bronchial artery angiography further demonstrated arterial malformation, tortuosity, and hypertrophy at the lesion site, consistent with a diagnosis of BDD.

**Interventions:**

The patient successfully underwent BAE via a left distal radial artery puncture.

**Outcomes:**

The patient’s lesion was utterly resolved during the 1-year follow-up, with no disease progression.

**Lessons:**

Clinicians should consider BDD in cases of unexplained hemoptysis. BAE is the preferred treatment, and if conventional transfemoral access is not feasible, the radial artery can serve as an alternative approach. This case provides practical support for diversifying interventional techniques in BAE.

## 1. Introduction

**Figure 1.**
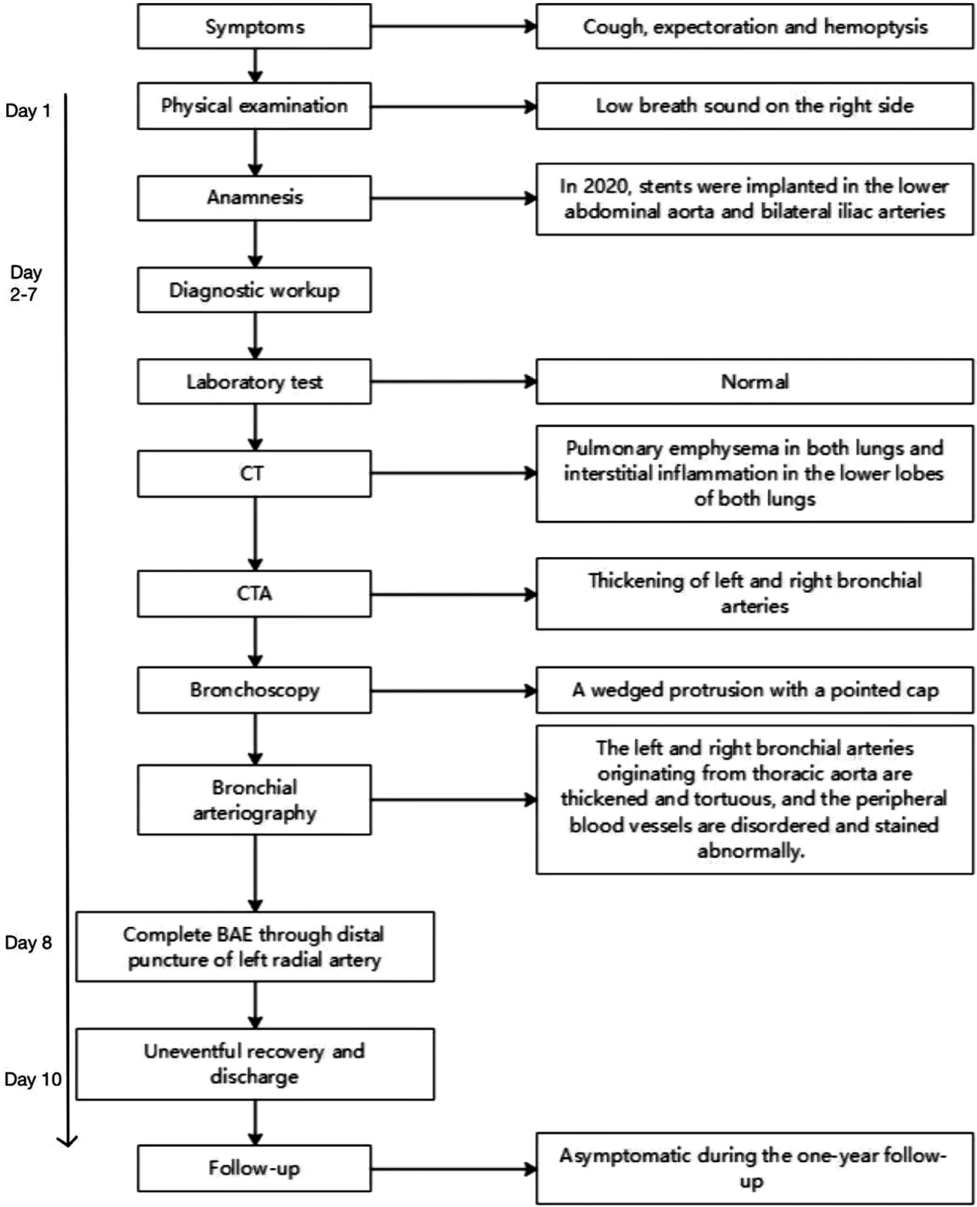
Timeline illustrating the diagnosis and treatment process of the patients. CT = computed tomography, CTA = computed tomography angiography, BAE = bronchial artery embolization.

Dieulafoy disease is a common disorder causing gastrointestinal bleeding, but it rarely appears in the bronchi. Daboussi et al^[1]^ collected 90 cases from January 1995 to December 2022 and concluded that the male-to-female ratio was 1.6; the incidence was higher in middle-aged and older adults; and the lesions were mostly located in the right bronchus. Here, we report a case of a patient with bronchial Dieulafoy disease (BDD) who presented with repeated hemoptysis and underwent bronchial artery embolization (BAE) for treatment (Fig. 1).

## 2. Case report

**Figure 2.**
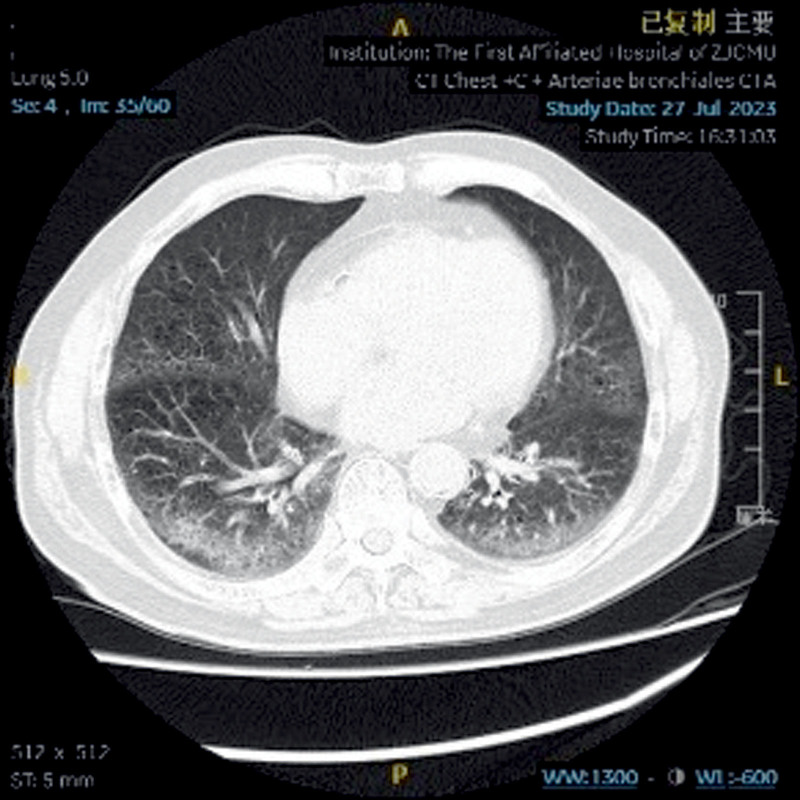
Plain chest computed tomography (CT) scan indicated pulmonary emphysema in both lungs and interstitial inflammation in the lower lobes of both lungs.

**Figure 3.**
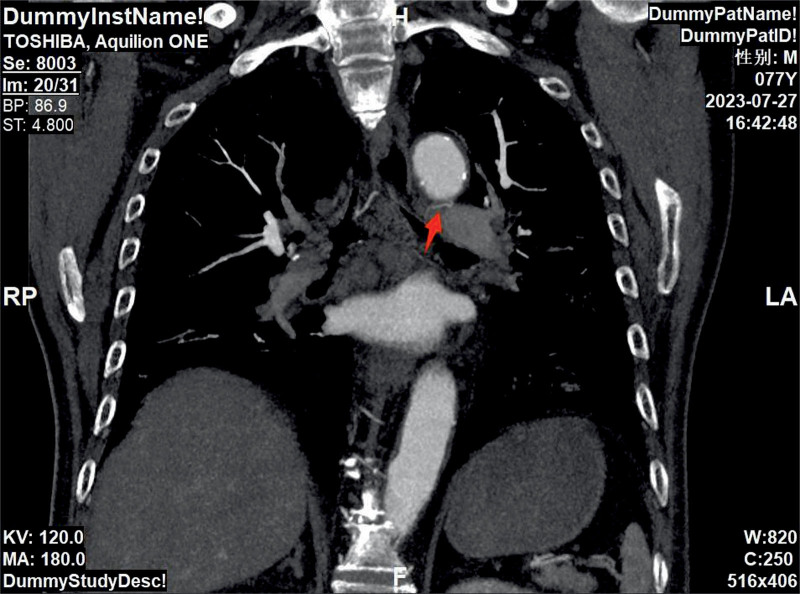
Computed tomography angiography (CTA) of the pulmonary artery showed thickening of the left bronchial artery, with the abnormal vessel marked by an arrow.

**Figure 4.**
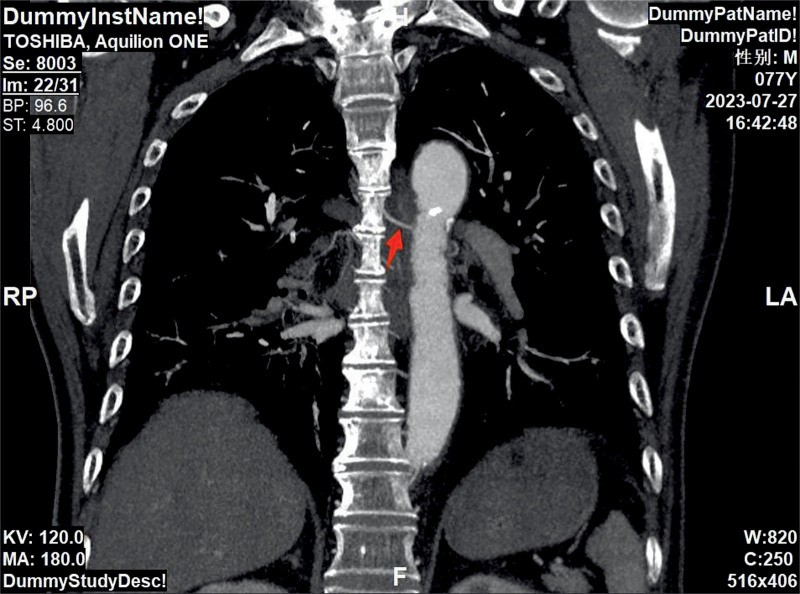
Computed tomography angiography (CTA) of the pulmonary artery showed thickening of the right bronchial artery, with the abnormal vessel marked by an arrow.

**Figure 5.**
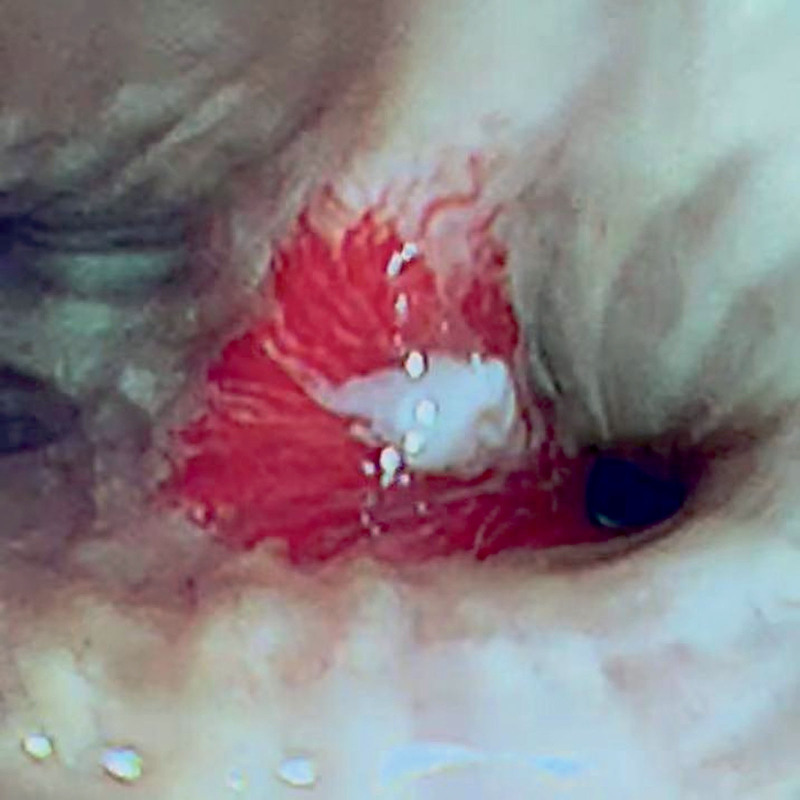
Bronchoscopy showed that at the opening of the ridge of the left upper lobe of the lung, there were crater-like concentrated mucosal blood vessels, and a white necrotic ulcer surface could be seen among them.

**Figure 6.**
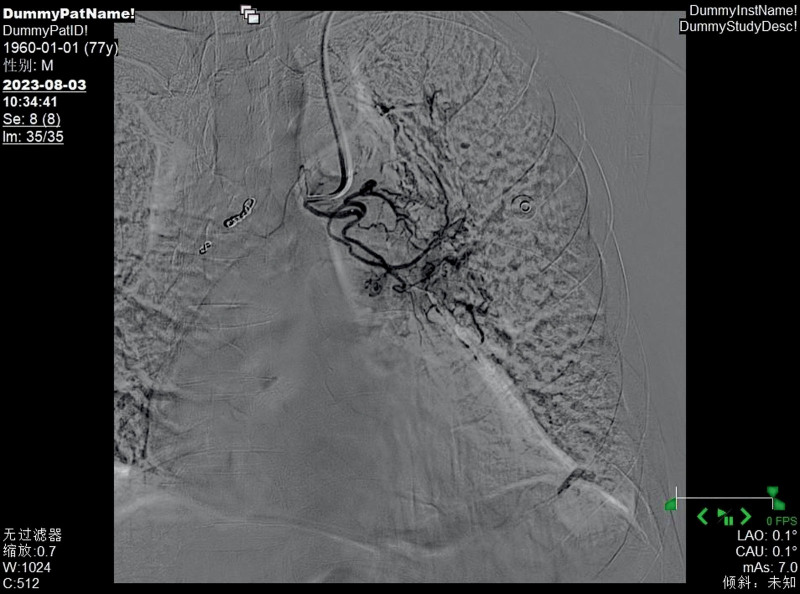
The left bronchial artery was thickened and tortuous, with disordered terminal blood vessels and abnormal staining.

**Figure 7.**
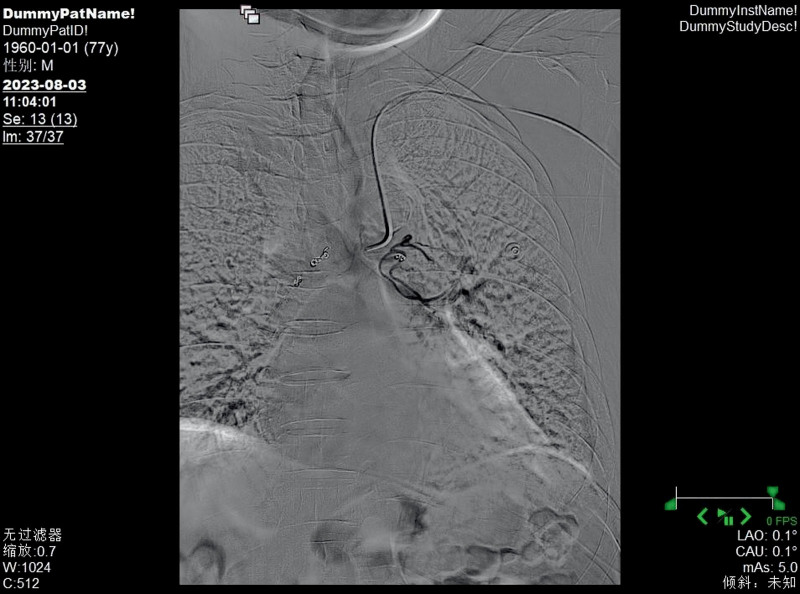
Left bronchial artery embolization therapy was carried out, and the disordered terminal blood vessels disappeared.

**Figure 8.**
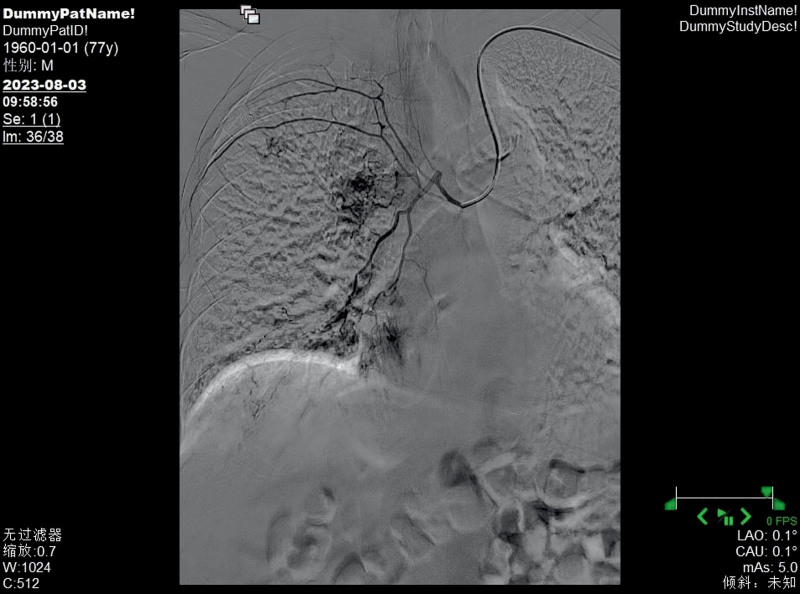
The right bronchial artery was thickened and tortuous, with disordered terminal blood vessels and abnormal staining.

**Figure 9.**
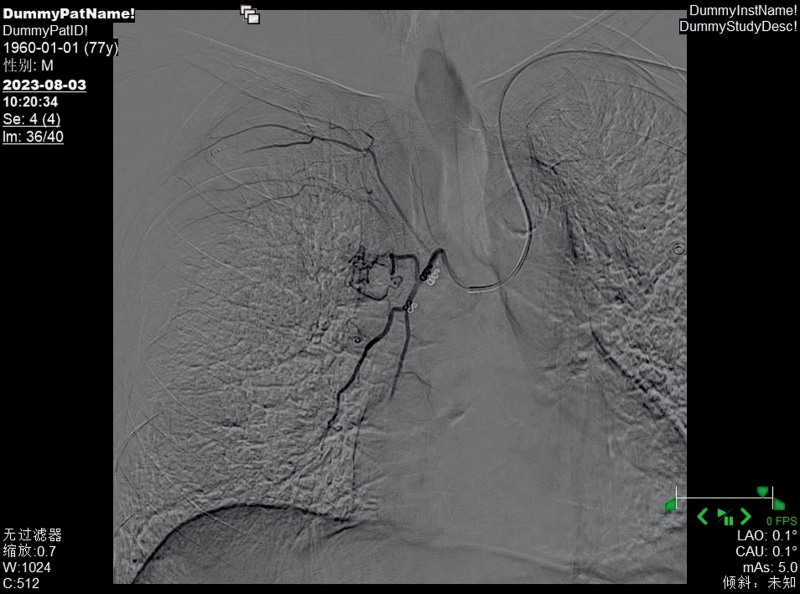
Right bronchial artery embolization therapy was carried out, and the disordered terminal blood vessels disappeared.

**Figure 10.**
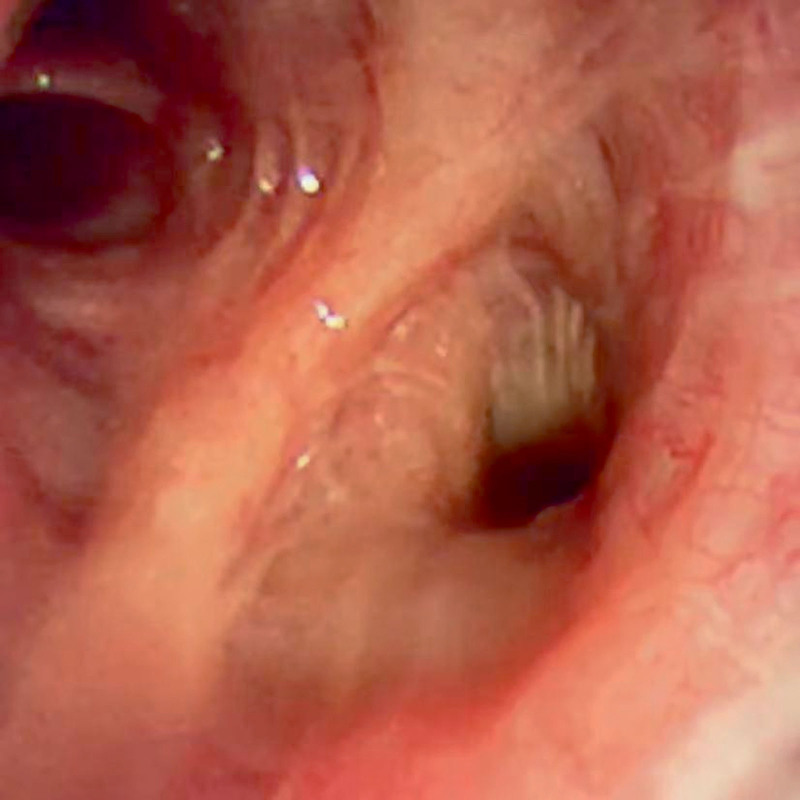
Bronchoscopy reexamination 1 month later showed that the lesions had disappeared entirely.

A 77-year-old male patient was admitted to The First Affiliated Hospital of Zhejiang Chinese Medical University on July 27, 2023, due to “cough, expectoration, and hemoptysis for 15 years, aggravated for >1 month.” The patient had a 60-year smoking history. For 15 years, the patient had experienced recurrent hemoptysis, and the symptoms were aggravated >1 month ago, accompanied by stuffiness in the right anterior chest area. In 2020, the patient underwent stent implantation in the lower segment of the abdominal aorta and bilateral iliac arteries due to intermittent claudication of both lower limbs and had to take aspirin for anticoagulation. However, due to the increased frequency and amount of hemoptysis, the drug was discontinued half a year later. The patient was in a dilemma. He was unsure whether to continue taking aspirin for anticoagulation or stop taking it to reduce the frequency of hemoptysis. Physical examination on admission showed that the breath sound on the right side was relatively low, and no abnormalities were found in the heart and abdomen examinations. Chest plain computed tomography (CT) scan indicated pulmonary emphysema in both lungs and interstitial inflammation in the lower lobes of both lungs (Fig. 2). CT angiography of the pulmonary artery showed thickening of the left and right bronchial arteries (Figs. 3 and 4). Under bronchoscopy, scattered exposed blood vessels were seen in the lumens of both lungs, and exposed mucosal blood vessels, vascular malformations, and a white ulcer surface in the middle were found in the upper lobe of the left lung (Fig. 5), suggesting BDD. However, the patient, in this case, had undergone stenting of the lower abdominal aorta-both iliac arteries, and it would be too risky to puncture through the femoral artery, which, in turn, added to the difficulty of treatment. In order to solve the symptoms of hemoptysis, bronchial artery angiography and embolization were completed through left distal radial artery puncture on August 3, 2023 (Figs. 6–9). One month later, bronchoscopy was reexamined, the lesions had completely disappeared (Fig. 10), and there was no hemoptysis during the 1-year follow-up.

## 3. Discussion

In 1995, Sweerts et al^[2]^ first described BDD, and its clinical manifestation was massive hemoptysis of unknown cause. Chronic bronchial inflammatory injury and congenital vascular malformations were considered to be the main causes of this disease.^[3]^ According to literature reports, recurrent hemoptysis is the primary clinical manifestation of BDD.^[4–6]^ Bronchoscopy and bronchial arteriography have important diagnostic value. Given the advantage of minimally invasive, BAE is the preferred treatment method.^[7]^ If this treatment method fails, lobectomy can be considered.^[8]^ In this case, the patient had repeated hemoptysis, but the chest CT scan could not rule out other diseases that could cause hemoptysis. Therefore, we performed a CT angiography of the pulmonary artery, which indicated thickening of the left and right bronchial arteries. To further clarify the diagnosis, we performed a bronchoscopy, and the results highly suggested BDD, so a biopsy was not performed. Finally, we innovatively completed the BAE by performing radial artery puncture, and the patient had no recurrence of hemoptysis within 1 year of follow-up.

Under bronchoscopy, BDD is often manifested as nodular protrusions of the mucosa,^[9,10]^ similar to polypoid new growth protrusions, and is easily misdiagnosed as tumors or other new growths and biopsied, which may lead to massive hemorrhage. Sometimes, it can also manifest as only clots, blood, or bleeding points.^[8–11]^ When lesions with the above characteristics are found during bronchoscopy, clinicians should pay attention to them, handle them carefully, and not perform a biopsy when BDD is suspected. Otherwise, it will cause massive hemorrhage in patients and seriously threaten their lives. If bleeding occurs after touching or biopsy, hemostatic measures must be quickly initiated to ensure the smoothness of the airway; if the situation is critical, emergency lung resection can be performed promptly to avoid serious consequences caused by bleeding. Zhou et al^[12]^ once reported a case in which a patient had a massive hemorrhage after a biopsy to exclude lung cancer. Despite emergency rescue and BAE, the patient could not be saved, and BDD was finally diagnosed by autopsy. Pathologically, it often presents as arterial malformations under the bronchial mucosa, where tortuous and dilated arteries form small nodules protruding into the bronchial lumen, and sometimes, the openings of the malformed blood vessels can be seen in the bronchial lumen.^[3,12,13]^

Bronchial arteriography and embolization are important diagnostic and treatment methods for BDD. Bronchial artery angiography can show arterial malformations, tortuosity, and thickening at the lesion site and is sometimes accompanied by bleeding signs.^[14]^ Whether these abnormal blood vessels are congenital, acquired, or just variations of normal blood vessels has not yet been determined.^[15]^ The bronchial artery originates typically from the proximal part of the descending aorta, but variations in the origin part are relatively common. Arteries originating from other parts of the aorta or vascular systems are called ectopic arteries.^[16]^ Choi et al^[17]^ recruited 600 patients and found that abnormal bronchial arteries most commonly originated from the aortic arch. Fang et al^[18]^ found that in addition to the bronchial artery, some branches directly originating from the pulmonary artery might abnormally open into the bronchial lumen. Through retrospective analysis of 17 patients, they found that 9 cases were from the bronchial artery and 3 cases were from the pulmonary artery. BAE can treat hemoptysis caused by bronchial artery injury for various reasons, block the blood supply of thoracic tumors, and treat bleeding from thoracic wall sinus tracts. The general operation process is to insert a catheter through the femoral artery, slowly advance the tube until the target blood vessel is found, inject a contrast agent to understand the degree of the lesion site, and inject embolic materials to block the blood supply of the artery at that site. Wang et al^[19]^ successfully controlled the hemoptysis symptoms of patients with BDD by this method, and there was no recurrence during the 1-year follow-up.

In this case, the patient received selective BAE after ineffective internal medicine treatment, and the hemoptysis symptoms were relieved. Its particularity lies in the fact that the patient had previously undergone stent implantation from the lower segment of the abdominal aorta to the bilateral iliac arteries, and the risk of femoral artery puncture was too high, which increased the difficulty of treatment. In 1992, Kiemeneij et al^[20]^ first selected the radial artery as the access route for coronary artery intervention therapy and achieved success. Since then, radial artery access has become an important complement to the femoral artery access technique. However, the use of radial artery access alone to treat bronchial feeding arteries is relatively rare. The primary reason is that compared to other commonly used interventional vessels, the radial artery has a smaller diameter. This anatomical characteristic, on the one hand, increases the difficulty of puncture, making it challenging for beginners and raising the likelihood of vascular spasm due to multiple attempts; on the other hand, it also elevates the risk of postprocedural vascular occlusion. Therefore, it is essential to perform the Allen test routinely before the procedure. In addition, the catheter must navigate through the aortic arch to reach the bronchial artery, further increasing the technical complexity of the operation. The results of the Allen test for both hands of this patient were positive, meeting the surgical conditions, and the surgery could be performed. Ultimately, we performed a puncture through the left distal radial artery, and the catheter was superselectively advanced into the left and right bronchial arteries. Angiography revealed hypertrophy and tortuosity of the bronchial arteries, disordered peripheral vessels, and abnormal staining, consistent with most literature reports. After superselective catheterization of the left and right bronchial arteries with a microcatheter, an appropriate amount of blank microspheres and coils were used for embolization. Repeat angiography showed satisfactory embolization, with the disordered peripheral vessels mainly disappearing. After BAE, the patient no longer had hemoptysis. One month later, a reexamination by bronchoscopy indicated that the lesions had disappeared. Up to this point, the patient’s dilemma, whether to take anticoagulant drugs to prevent thrombosis or stop taking them to reduce the frequency of hemoptysis, was successfully resolved, and the patient could take anticoagulant drugs without worry. In the medical field, traditional BAE is usually performed by puncturing the femoral artery. However, some patients have contraindications for femoral artery puncture, which affects the implementation of the operation. At this time, another access route is needed as a substitute. Moreover, the radial artery has advantages such as being easy to compress for hemostasis after surgery, not requiring bed rest, and having a fast recovery. This case has provided a new interventional approach option for BAE, which helps to promote the diversified development of interventional techniques.

In order to further conduct statistical induction on the previously reported cases of BDD, we used “bronchial” and “Dieulafoy” as keywords and conducted searches in the Wanfang database, the VIP database, the China National Knowledge Infrastructure, and the Full-Text Database of Chinese Medical Journals. We also used “bronchial Dieulafoy disease” as the search term in the All Fields of the PubMed database for relevant literature retrieval. A total of 77 articles were retrieved. After deleting duplicate reports, conference papers, articles with incomplete information, and nursing literature, a total of 68 articles were included, reporting 107 cases of BDD. Among them were 70 male cases and 37 female cases, with ages ranging from 9 months to 82 years. The most common symptom was repeated hemoptysis (85.98%, 92 cases). Among them, 26 patients had mucosal biopsies, and bleeding occurred; 7 patients stopped bleeding after BAE, 7 patients stopped bleeding after resection of the affected lobe of the lung, and another 8 patients died due to ineffective rescue from massive hemorrhage. The situation of literature retrieval is shown in Table 1. Therefore, further research on this disease is still needed in clinical practice.

**Table 1:** Summary table of the retrieved literature on bronchial Dieulafoy disease.

Classification	Performance
Age	Ranging from 9 months to 82 years old, with the disease onset being more common in middle age.
Gender	There were 70 male cases and 37 female cases.
Clinical manifestations	The most common manifestation was repeated hemoptysis (85.98%, 92 cases). It could also present as cough (23.36%, 25 cases), dyspnea (3.7%, 4 cases), chest pain (1.9%, 2 cases), repeated infections (0.9%, 1 case), and so on.
Lesion sites	There were 74 cases in the right lung, among which 6 cases were in the right main bronchus, 4 cases in the intermediate bronchus, 11 cases in the right upper lobe, 20 cases in the right middle lobe, and 36 cases in the right lower lobe. There were 36 cases in the left lung, among which 10 cases occurred in the left main bronchus, 14 cases in the left upper lobe, and 11 cases in the left lower lobe. There were 4 cases with simultaneous occurrence in both lungs and 1 case in the tracheal carina.
Bronchoscopy	Bronchoscopy was performed on 105 cases. In 72 cases, nodular neoplasms were visible inside the bronchi, with smooth or rough surfaces that were congested, and some of the surfaces were covered with white secretions (9 cases). In 7 cases, massive bleeding or blood clot formation was seen in the lumen of the affected lobe or segment. In 3 cases, mucosal ulcers were observed at the lesion sites. In 3 cases, tortuous submucosal blood vessels could be seen. In 2 cases, earthworm-like elevations of the mucosa were noted. There was no description for 18 cases.
Prognosis of bronchoscopic biopsy	Twenty-six patients underwent mucosal biopsy and bleeding occurred. After local hemostasis, bleeding stopped in 3 cases. One case achieved hemostasis by ligation of abnormal blood vessels. Seven cases stopped bleeding after BAE, and 7 cases stopped bleeding after resection of the affected lobe of the lung. Another 8 patients died due to ineffective rescue from massive hemorrhage.
Treatment	Seventy-four patients were treated with BAE. Twenty-seven patients underwent resection of the affected lobe of the lung. Among them, 15 patients had their treatment changed to lobectomy due to unsuccessful embolization. Three patients underwent bronchoscopic argon plasma coagulation. Two patients underwent laser ablation, and 1 patient underwent electrocautery ablation.

BAE = bronchial artery embolization.

## 4. Conclusion

BDD is a relatively rare vascular abnormality disease. Although the number of reports on this disease is gradually increasing, its etiology, pathogenesis, and diagnostic criteria are still unclear and need further in-depth exploration by scholars. In this case, given that the patient did not meet the conditions for femoral artery puncture, it was ultimately decided to perform bronchial arteriography via radial artery puncture. This decision not only successfully resolved the patient’s angiography problem, but, more importantly, it provided a practical alternative for patients who, in the future, also do not meet the conditions for femoral artery surgery, thus broadening the clinical diagnosis and treatment approach.

## Acknowledgments

The authors are grateful to the patient for agreeing to post his information and to all the medical staff.

## Author contributions

**Conceptualization:** Jiabin Qian, Yiheng Qian.

**Data curation:** Jiabin Qian, Xin Lv.

**Formal analysis:** Jiabin Qian, Xin Lv.

**Writing – original draft:** Jiabin Qian.

**Methodology:** Ruilin Chen.

**Project administration:** Ruilin Chen.

**Funding acquisition:** Xin Lv.

**Writing – review & editing:** Xin Lv.
